# Perceiving reduced physical activity during COVID-19 lockdown is related to lower quality of life: a cross-sectional study with young adults

**DOI:** 10.1007/s12662-021-00795-7

**Published:** 2022-01-24

**Authors:** Christina Niermann, Lukas Bollenbach, Martina Kanning

**Affiliations:** Department of Sport Science, University of Konstanz, Konstanz, Germany

**Keywords:** Mental health, Well-being, Sports, Exercise, Pandemic

## Abstract

Physical activity and social participation are positively related to mental health and represent resources that strengthen individuals’ resilience. However, the measures aiming to contain the coronavirus disease 2019 (COVID-19) pandemic included restrictions regarding these health resources. For many people, stay-at-home orders had a negative effect on mental health and health-related behaviors such as physical activity. Young adults seem to be a particularly vulnerable group. The study aimed to examine the relationship between changes in physical activity and perceived quality of life in young adults during lockdown conditions. We conducted an online survey during the second lockdown in Germany and assessed perceived changes in physical activity, social activities, quality of life (QoL), the perceived burden of changes in social activities, and current QoL in 212 young adults (63.7% women, mean age = 23.46, standard deviation = 3.63). Young adults that maintained or increased their physical activity level under lockdown conditions, rated their current QoL higher and perceived a less negative change of their QoL during lockdown compared to those with decreased physical activity. Furthermore, those who rated that their physical activity levels did not change or increased, perceived the reduction of their social activities as less burdening. The results indicate that physical activity is a beneficial health resource during lockdown conditions. This highlights the importance of gaining knowledge regarding the antecedents of reducing physical activity and developing strategies that support young adults to be physically active in challenging times such as the pandemic (e.g. ehealth/mhealth approaches).

## Introduction

Being physically active and participating in social activities are positively related to mental health (Lee, Jang, Lee, Cho, & Park, 2008; Rebar et al., 2015) and represent important health resources (Biddle, Mutrie, Gorely, & Faulkner, 2021; Lee et al., 2008). However, the measures aiming to contain the coronavirus disease 2019 (COVID-19) pandemic included restrictions, especially regarding these health resources. During the lockdown social contacts were supposed to be reduced to a minimum; participating in social activities was not allowed, and sports clubs, fitness centers, etc. were closed. This study aimed to examine the association of perceived changes in physical activity levels with quality of life (QoL) and social activities (SA) during the second lockdown in Germany in young adults.

Reducing social contacts affects mental health, including depression, anxiety, and loneliness (Leigh-Hunt et al., 2017). Furthermore, repeated daily life stressors, such as problems juggling work and family demands during the lockdown, have a cumulative effect on health and well-being (Almeida, 2005). Accordingly, studies showed high levels of mental health problems, such as symptoms of anxiety, depression, and loneliness after the lockdown came into effect (Buecker et al., 2020; Daly, Sutin, & Robinson, 2020; Mata et al., 2021; Niedzwiedz et al., 2021).

However, the lockdown did not equally affect all people. For example, Tušl, Brauchli, Kerksieck, and Bauer (2021) found that 13% of their participants perceived an improvement in their private lives, which was related to higher mental well-being. Several studies indicated that young adults (< 35 years) were particularly affected by the COVID-19 pandemic and lockdown measures. Overall, for this age group the highest increases in mental health problems have been found (Daly et al., 2020; Niedzwiedz et al., 2021; Pieh, Budimir, & Probst, 2020; Tušl et al., 2021). Furthermore, Naughton et al. (2021) showed that younger age was associated with negative health behavior changes, being younger was associated with higher reductions of days with 30 min or more of self-reported moderate to vigorous physical activities. Therefore, the relevance of health resources in young adults becomes evident. Health resources are protective factors, that strengthen physical and mental health and enable individuals to cope with internal or external demands (Becker, 2003; Reimann & Hammelstein, 2006). Physical activity (PA) and social participation are health resources (Biddle et al., 2021; Lee et al., 2008). For instance, they have the potential to buffer the effect of stress and it has been shown that they are related to mental health and quality of life (Berger & Tobar, 2007; Klaperski, 2018; Lakey & Orehek, 2011; Rebar et al., 2015; Smith, Hill, & Kokanovic, 2015).

The restrictions aimed to inhibit social contacts and thereby instructed sport and exercise did not take place, sports clubs and gyms were closed and access to areas for moving and recreation was limited. Current study results reflect these restrictions and found an overall decrease in PA and an increase in sedentary time (Mutz & Gerke, 2021; Naughton et al., 2021; Sidebottom, Ullevig, Cheever, & Zhang, 2021; Tison et al., 2020). However, results are inconsistent which might be partly due to differential effects of the restrictions on different PA domains: while sports club PA decreased, other leisure time PA such as jogging or hiking were not affected (Aegerter et al., 2021; Di Renzo et al., 2020). Further studies found interindividual differences; for some people or groups of people, PA levels decreased, while for others, PA levels maintained stable or increased (Fearnbach et al., 2021; Mutz & Gerke, 2021). A further explanation might be the time of the assessment during the lockdown as studies showed that after a decrease in PA at the beginning of the lockdown, PA levels increased two months later (Mata et al., 2021; Tison et al., 2020).

Even though sports clubs, gyms, etc. were closed, people were allowed to be physically active. Current studies indicate that being physically active is positively associated with mental health, well-being, and quality of life under the pandemic/lockdown conditions and can help to cope with the challenges arising from the pandemic situation and its restrictions in everyday life (Brailovskaia et al., 2021; Maher, Hevel, Reifsteck, & Drollette, 2021; Mata et al., 2021; Zach, Fernandez-Rio, Zeev, Ophir, & Eilat-Adar, 2021). In contrast, social activities, such as meeting family and friends, going to bars, etc., were not possible. This study aimed to examine the association between perceived changes in PA levels during lockdown conditions and current QoL, and perceived changes in QoL. We assumed that maintaining or increasing PA levels during the lockdown period is associated with a better rating of current QoL and a less negative change in QoL. Furthermore, we explored the associations to perceived changes in social activities (SA) and to the perceived burden of SA reduction.

## Methods

### Study design

A cross-sectional online survey was conducted using the German platform SoSci Survey (https://www.soscisurvey.de/; SoSci Survey GmbH, Munich, Germany). After pilot testing the items, data were collected between 18 January 2021 and 28 February 2021, in the middle of the second “hard” lockdown in Germany (December 2020–April 2021). Lockdown restrictions varied, but consisted of social distancing, including reduction of social contacts, and the closure of social and cultural institutions (e.g. restaurants, cinema, museums) and places where PA can usually take place (e.g. gyms, pools). The first page of the questionnaire contained a brief description of the study aims. Participants gave informed consent by indicating that they had read and understood the study information and were willing to participate. It took about 10 min to complete the questionnaire.

### Procedure and participants

Dissemination of the link to the online survey took place via social media (Facebook, Instagram; Meta Platforms, Inc., Menlo Park, CA, USA). To reach our target population, university students and other young adults were asked to follow the Instagram and Facebook accounts that were created and help to disseminate the survey itself (snowball sampling) and information about it. Information on the study was regularly posted and consisted of pictures and infographics with concomitant information, the survey link, and open questions to initiate reader engagement. Furthermore, accounts with high numbers of followers were asked to repost or link the survey, and relevant accounts (e.g. local newspaper, student union) were linked. Participants could voluntarily opt-in to participate in a raffle for 10 vouchers each with a value of 20 € for local stores.

### Measures

Considering compliance, we kept the questionnaire as short as possible and used single-item measures. Single-item measures for self-evaluated health and QoL are widely used and well-established (Ahmad, Jhajj, Stewart, Burghardt, & Bierman, 2014). The questionnaire was in German.

Besides demographics, the following variables were assessed:

1. Perceived change of PA: “Do you currently experience your sports and physical activities (e.g. walking, biking, gardening, jogging, home exercise) as changed?”,
2. Perceived change of SA: “Do you currently experience your social activities (shopping, meeting family/friends/acquaintances, pursuing hobbies outside your own four walls, etc.) as changed?”,
3. Perceived change of QoL: “To what extent do you currently experience your quality of life as changed?”,
4. Perceived burden of SA changes: “How burdening is it to you?”,
5. QoL: “How would you rate your quality of life?”.

(1)–(3) were answered on 5‑point Likert scales (−2 = much less to 2 = much more), (4) was answered on a 4-point Likert scale (1 = not at all to 4 = very much) (only answered when (2) was answered with “much less” or “somewhat less”), and (5) was answered on a 5-point Likert scale (1 = very bad to 5 = very good).

### Analyses

Data were analyzed using SPSS 27.0 (IBM Corp., Armonk, NY, USA). Missing values were excluded pair-wise. Due to the violation of normal distribution of the data, we used bootstrapping to obtain estimates of the standard errors and compute confidence intervals and significance tests. For calculating bias-corrected 95% confidence intervals, 1000 bootstrapping iterations were requested.

## Results

### Descriptive characteristics

The mean age of the 212 participating young adults (63.7% female) was 23.46 years (*SD* = 3.63). Our sample was highly educated; more than 90% had a university-entrance diploma (‘Abitur’) or an advanced technical college certificate (‘Fachhochschulreife’). While 104 (51%) individuals stated that they reduced their PA during lockdown conditions, 100 individuals perceived their PA as equal (38, 18.6%) or higher (62, 30.4%) than usual. Perceived changes in PA are significantly associated with perceived changes in social activities (SA), perceived burden of SA changes, current perception of quality of life (QoL), and perceived changes in QoL. Effect sizes were small. The descriptive statistics and correlation coefficients for study variables are shown in Table 1.

**Table 1:** Descriptive characteristics and correlation coefficients for study variables

			Pearson correlation coefficients^a^ *r* (*p*), (CI)			
Variables		*M* (*SD*), *n*	2	3	4	5
1	Perceived change in PA^b^	−0.39 (1.22), 204	0.15 (0.04), (0.003, 0.28)	−0.16 (0.02), (−0.30, −0.16)	0.20 (0.005), (0.06, 0.33)	0.15 (0.03), (0.01, 0.29)
2	Perceived change in SA^b^	−1.71 (0.76), 211	–	−0.37 (< 0.001), (−0.50, −0.21)	0.06 (0.43), (−0.09, 0.20)	0.23 (0.001), (0.06, 0.39)
3	Perceived burden of SA reduction^c^	3.26 (0.75), 200	–	–	−0.05 (0.51), (−0.20, 0.10)	−0.41 (< 0.001), (−0.52, −0.27)
4	Quality of life^d^	3.70 (0.98), 208	–	–	–	0.21 (0.003), (0.05, 0.36)
5	Perceived change QoL^e^	−1.03 (0.75), 207	–	–	–	–

*SD* standard deviation, *M* mean, *CI* 95% confidence interval, *PA* physical activity, *SA* social activities, *QoL* quality of life

^a^based on bias-correcting bootstrapping with 1000 samples

^b^values ranging from −2 = much less to 2 = much more

^c^values ranging from 1 = not at all to 4 = very much

^d^values ranging from 1 = very bad to 5 = very good

^e^values ranging from −2 = considerably worsened to 2 = considerably improved

No gender specific differences were found for perceived change in PA, perceived change in SA, QoL, and perceived change in QoL (*p*s = 0.18–0.93). However, women perceived a higher burden of the reduction of SA (*t*[194] = 2.46, *p* < 0.02, *d* = 0.60).

### Correlates of perceived changes in PA

Comparing young adults that perceived a negative change in their PA level (performing much less and less PA than before the pandemic) and people that stated that they maintained or increased their prepandemic PA levels revealed differences of moderate effect sizes in current QoL, and perceived changes in QoL. Furthermore, we found that the groups did not differ significantly regarding their perceived change of social activities, whereas the perceived burden of the compelled reductions of social activities was significantly lower in those who perceived no changes or an increase of their PA level (Table 2).

**Table 2:** Comparison of means between persons perceiving a decrease vs. no change or increase of physical activity (PA) during lockdown

	Decrease of PA	No change–increase of PA		
	*M* (*SD*)	*M* (*SD*)	*t* (*p*), *SE* (CI)^a^	Cohen’s *d*
Quality of life^b^	3.54 (1.07)	3.88 (0.91)	−2.40 (0.02), 0.14 (−0.63, −0.07)	0.60
Perceived change QoL^c^	−1.18 (0.81)	−0.93 (0.81)	−2.41 (0.02),0.11 (−0.46, −0.05)	0.59
Perceived change in SA^d^	−1.90 (0.30)	−1.82 (0.38)	−1.54 (0.14), 0.05 (−0.18, 0.02)	0.23
Perceived burden of SA reduction^d^	3.38 (0.68)	3.16 (0.80)	2.05 (0.05), 0.11 (0.02, 0.43)	0.58

*SD* standard deviation, *M* mean, *SE* standard error, *CI* 95% confidence interval, *PA* physical activity, *QoL* quality of life, *SA* social activities

^a^based on bias-correcting bootstrapping with 1000 samples, SE of mean differences and bias-corrected confidence intervals for mean differences are depicted

^b^values ranging from 1 = very bad to 5 = very good

^c^values ranging from −2 = considerably worsened to 2 = considerably improved

^d^values ranging from −2 = much less to 2 = much more

^e^values ranging from 1 = not at all to 4 = very much

## Discussion

In this study, we examined how perceived changes in PA levels during lockdown conditions are related to QoL. Data were collected during the second lockdown in Germany and focused on young adults as previous research has shown that this age group is particularly affected by COVID-19 restrictions. Younger adults that maintained or increased their PA level despite the restrictions, rated their QoL higher and perceived a less negative change of their QoL after the measures came into effect. Furthermore, they perceived the reduction of their social activities as less burdening.

In line with current studies, we found interindividual differences, the restrictions differently affected individuals’ well-being and health-related behaviors (Tušl et al., 2021). Half of our sample maintained or increased their PA level during the lockdown. Together with other studies, mostly from the first lockdown beginning in March 2020, the results indicate that changes in PA levels might be relevant for mental health outcomes. Zach et al. (2021) found that people who were physically active during the lockdown period reported a higher level of resilience and positive feelings, and a lower level of depression, compared with those who were not physically active. Furthermore, they found that changes in PA habits from being active prelockdown to becoming inactive during lockdown were related to increased depressive symptoms. Maher et al. (2021) found that PA is positively associated with college students’ positive affect in young adults and that the change in minutes of PA (before vs. during COVID stay-at-home orders) was positively associated with a change in positive affect: the higher the decrease in PA, the larger the decrease in positive affect. In addition, we found a link between perceived changes in PA and SA, i.e. persons who perceived no changes or increased PA levels evaluated the reduction of their social activities as less severe. Furthermore, the more burdening the persons rated the reduction of SA, the more negative they perceived their QoL changes. These results are in line with research highlighting the role of social participation for an individual’s mental health (e.g. Leigh-Hunt et al., 2017). Together with findings from, for example, Flueckiger, Lieb, Meyer, Witthauer, and Mata (2016) who found a stress-buffering effect of PA in two intensive longitudinal studies, these results indicate that PA is a health resource that is beneficial in times that imply challenges or stressful events.

The main strength of our study is that we gathered data of a particularly vulnerable age group—young adults—in the second lockdown in Germany. We focused on changes in PA levels due to the restrictions. However, according to our recruitment strategy (social media) and in terms of compliance, we kept the questionnaire as short as possible and used single-item measures. This study is based on cross-sectional data. Therefore, assumed causalities have to be verified by longitudinal studies and experiments. Furthermore, our sample was higher educated than the average German population of young adults which possibly limits the generalizability.

## Conclusion

Perceiving no changes or an increase of PA levels during the lockdown is related to higher QoL. The results underline the importance of not only promoting PA in normal life but even more in challenging times such as the pandemic. This includes the development of targeted strategies to support young adults to be physically active in such challenging times (e.g. ehealth/mhealth approaches). Further studies should focus on predictors (e.g. self-determination, habit strength) of changing vs. maintaining health behaviors following stressful life events such as the pandemic situation and its necessary restrictions.

## Declarations

The study fully conformed to the Declaration of Helsinki and the ethics guidelines of the German Psychological Society. Informed consent was given (via check box) prior to the beginning of the online survey. Full ethical approval for the AMbit project was obtained from the University of Konstanz (IRB18KN010-004, October 29, 2018).
